# Reviewer’s List

**DOI:** 10.1109/JTEHM.2021.3132409

**Published:** 2021-12-06

## Abstract

Our deepest gratitude to everyone who reviewed papers for JTEHM in 2020.

Our deepest gratitude to everyone who reviewed papers for JTEHM in 2020.

A Ahmad, Fayyaz
  Akcakaya, Murat
  Albuquerque, Victor Hugo C.
  Aliverti, Andrea
  Amukele, Timothy Kien
  Assba, Adnan
  Auffermann, William
  AuYeung, Michael
  Avolio, Alberto
B Barbaro, Massimo
  Baumert, Mathias
  Bekiroglu, Korkut
  Ben Salah, Ridha
  Bian, Gui-Bin
  Biswas, Dwaipayan
  Borkholder, David
  Bourke, Alan
  Brattain, Laura
  Burgio, Marco Dioguardi
C C. V., Aravinda
  Cadamuro, Janne
  Carek, Andrew
  Catherwood, Philip
  Chan, Jessica
  Chandra, Amit
  Charlton, Peter
  Chen, Liming
  Chen, Mei-Juan
  Clever, Henry M.
  Conway, Ted
  Cordella, Francesca
  Cuffe, Paul
D Davalos, Rafael
  David, Marcelo
  Dimeski, Goce
  Dimitrakakis, Emmanouil
  Dong, Lin
  Drotar, Peter
  Drummond, Colin
  Du, Xiuquan
E Eerikäinen, Linda
  Elze, Tobias
  Ema, Ryoichi
F Flood, Matthew
  Földi, Sándor
  Fregly, B. J.
G Gao, Wei
  Geng, Weidong
  Gillespie, Stephanie
  Göbel, Peter
  Gore, Russell
  Guidoboni, Giovanna
  Gutierrez, Guillermo
  Guvenis, Albert
H Haghayegh, Shahab
  Han, Dong
  Hassan, Rabab
  Hassan, Umer
  Hidayat, Wahyu
  Hossain, Md Ekramul
  Hughes, Alun D.
  Hwang, Ji-Young
I Inan, Omer
J Jackemeyer, David
  Jarrett, Francesca
  Jiang, Kui
  Jung, Christian
K Kamiński, Maciej
  Karisik, Filip
  Kassab, Mohamad
  Kau, Lih-Jen
  Kenyon, Nicholas
  Khan, Amir
  Khoo, Michael
  Kim, Chang-Sei
  Kim, Insoo
  Kingston, David
  Kopriva, Ivica
  Kuang, Shaolong
  Kumar Paul, Bikash
  Kwon, Richard
L Lambercy, Olivier
  Larson, Eric
  Lee, Jinseok
  Lee, Sunghoon
  Lendaro, Eva
  Li, Changsheng
  Li, Jingsong
  Li, John
  Liao, Hongen
  Lim, Miranda
  Lin, Chin-Sheng
  Liu, Pang-Yen
  Liu, Shuangjun
  Liu, Tao
  Long, Xi
  Lopez Valdes, Alejandro
M Mahmud, Tanvir
  Mankodiya, Kunal
  Mastinu, Enzo
  Mathew, Shawn
  Mathews, Robert
  Matsumura, Kenta
  Mazzoni, Alberto
  McKinnon, Brian
  Mendonça, Fabio
  Molenbroek, Johan
  Moore, Bria
  Morabito, Francesco
  Moreno, Courtney
  Mukesh, Sagarika
  Munoz, Camila
N Nunes, Catarina S.
P Panescu, Dorin
  Patel, Vishal
  Penzel, Thomas
  Peters, Terry
  Pethig, Ron
  Priye, Aashish
Q Qi, Jun
R Rahman, Md Awsafur
  Rajaraman, Sivaramakrishnan
  Ran, An-Ran
  Rao, Adam
  RaviChandran, Narrendar
  Reid Bush, Tamara
  Reilly, Richard
  Riaan, Stopforth
  Rocon, Eduardo
  Rong, Yibiao
  Roy Chowdhury, Shubhajit
  Rubinstein, Rebecca
  Rusz, Jan
S Sanford, Joseph
  Santosh, K. C.
  Sathyanarayana, Aarti
  Scassero, Matthew
  Semiz, Beren
  Sexton, Kevin
  Shao, Dangdang
  Shen, Huimin
  Sherratt, Robert
  Sinha, Sharad
  Song, Chao
  Sotoudeh, Houman
  Spadaro, Savino
  Spitzley, Kate
  Standaert, Francois
  Stoykov, Mary Ellen
  Stridh, Martin
  Sudirman, Sud
  Sugimachi, Masaru
  Suinesiaputra, Avan
T Töreyin, Hakan
  Torp, Hans
  Trabelsi, Hedi
  Trappey, Amy
  Traver, Vicente
  Tridandapani, Srini
  Tsow, Francis
U Urwyler, Prabitha
V Vehkaoja, Antti
  Vettoretti, Martina
  Vrajitoru, Dana
W Wallace, Bruce
  Wang, Chengjia
  Wang, Chonghe
  Wang, Shangying
  Wang, Shuo
  Wang, Xinggang
  Wang, Zhihui
  Wang, Zimo
  White, Ethan
  Wilson, David
  Witowski, Jan
  Wittenberg, Thomas
  Wu, C.
X Xian, Xiaojun
  Xu, Feng
Y Yang, Po
  Yang, Xu
  Yao, Jingting
  Yao, Yu
  Ye, Xujiong
  Ye, Yalan
  Yu, Hojeong
  Yu, Hui
Z Zhang, Guanqun
  Zhang, Xingyi
  Zhang, Yangsong
  Zhang, Zhilin
  Zhao, Ni
  Zheng, Xiaochen
  Zheng, Yushan
  Zwiggelaar, Reyer

